# Supplementary material for: In-sensor reservoir computing system for latent fingerprint recognition with deep ultraviolet photo-synapses and memristor array
Source: Nat Commun. 2022 Nov 3;13:6590. doi: 10.1038/s41467-022-34230-8 (PMC9633641; doi:10.1038/s41467-022-34230-8)
Supplement: Supplementary file 1 — Supplementary Information [file 41467_2022_34230_MOESM1_ESM.pdf]

Supplementary Information

In-sensor reservoir computing system for latent fingerprint recognition with deep ultraviolet photo-synapses and memristor array

Zhongfang Zhang, Xiaolong Zhao,* Xumeng Zhang,* Xiaohu Hou, Xiaolan Ma, Shuangzhu Tang, Ying Zhang, Guangwei Xu, Qi Liu, Shibing Long*

*Corresponding author. Email: xlzhao77@ustc.edu.cn; xumengzhang@fudan.edu.cn; shibinglong@ustc.edu.cn;

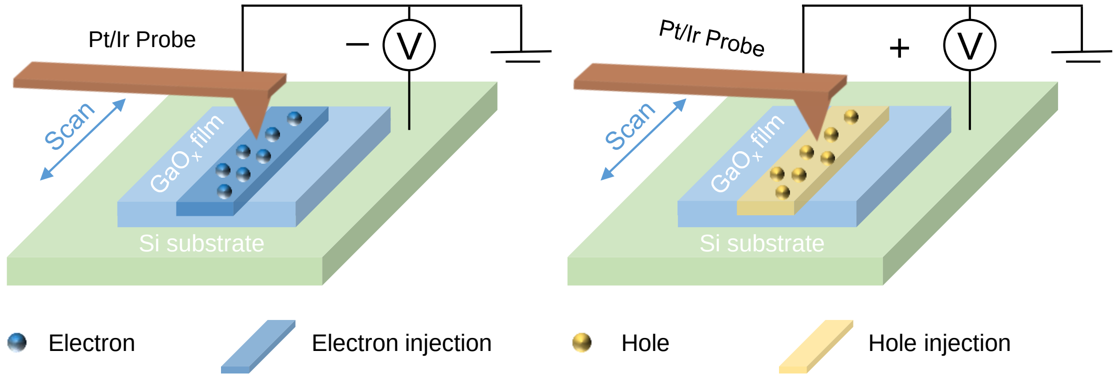

Supplementary Fig. 1 The schematic diagram of KPFM measurement of the a-GaO$_x$ film. The KPFM method reflects the potential distribution of the object surface by measuring the capacitance between the probe and the surface. Electrons or holes were injected into the film by negative (left panel) or positive (right panel) biasing during the scanning. In the target regions (5×5 μm^2), only the middle parts (1×5 μm^2) were injected with extrinsic electrons or holes.

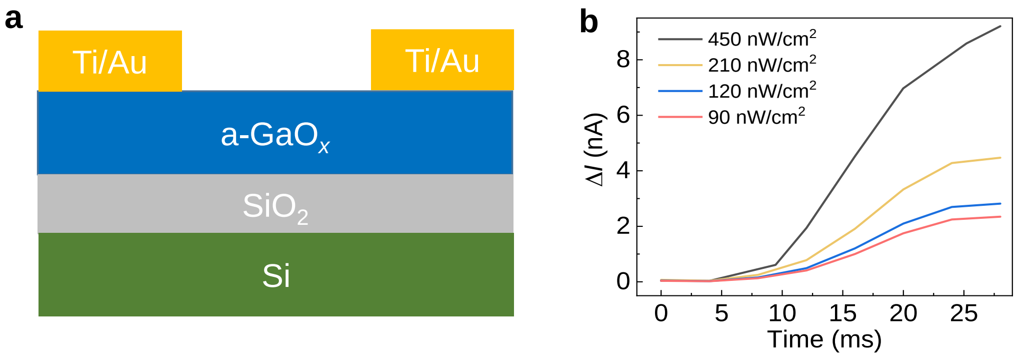

Supplementary Fig. 2 a-GaO$_x$ device and its nonlinear photoresponse. a Schematic diagram of the cross-section structure of the a-GaO$_x$ device. **b** Nonlinear dependence of ΔI on DUV light pulse width (25 ms) under various power densities.

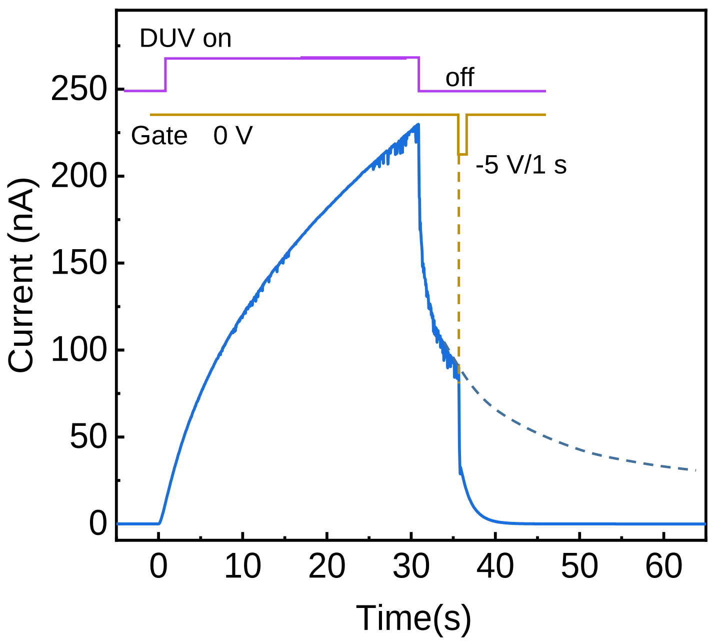

Supplementary Fig. 3 The erase of the photocurrent by negative gate voltage pulse. At any time during the decay of photocurrent, applying a negative pulse (*e.g.* -5 V/1 s) to the back gate can rapidly restore the current to the initial dark current.

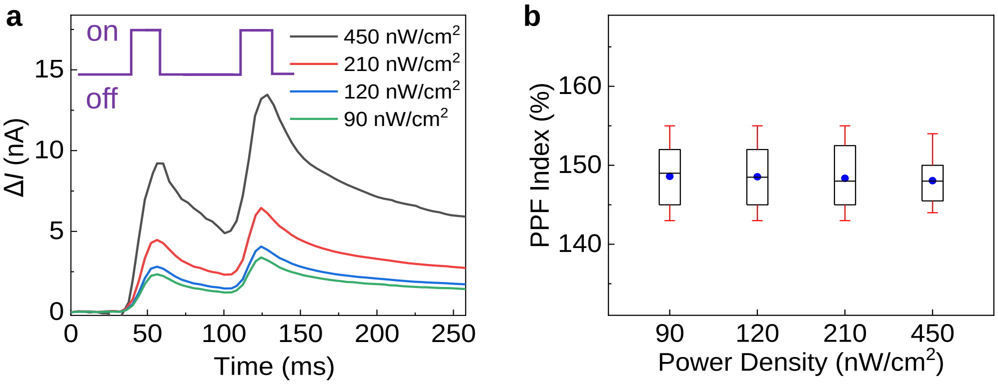

Supplementary Fig. 4 Influence of illumination densities on PPF. a Photoresponse I-t curves under various DUV power densities with the same pulse width (25 ms) and interval (45 ms). **b** Statistic distributions of PPF indexes extracted from 20 cycles for each illumination density. The minor fluctuation of PPF index indicates that power density has no obvious effect on the facilitation efficiency of this a-GaO_x photo-synapse.

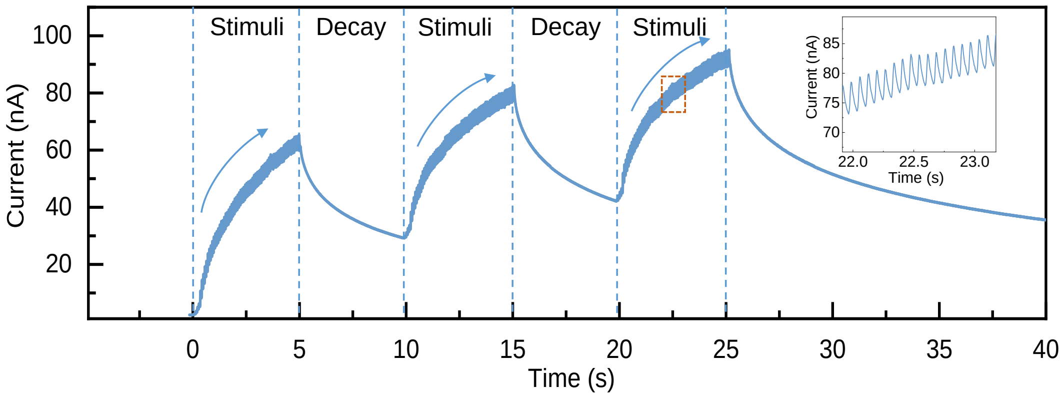

Supplementary Fig. 5 Nonlinearity photoresponse of the a-GaO$_x$ photo-synapse under multi-bit pulse stimuli. In each stimuli-decay cycle, the a-GaO$_x$ photo-synapse presents a nonlinearly modulable conductivity when suffering 72 DUV pulse stimuli. The photoresponse to stimuli always maintains nonlinearity in all cycles before the saturation of response current. The inset shows the close-up view of the brown dashed box, demonstrating the consectutive stimuli within 1 s.

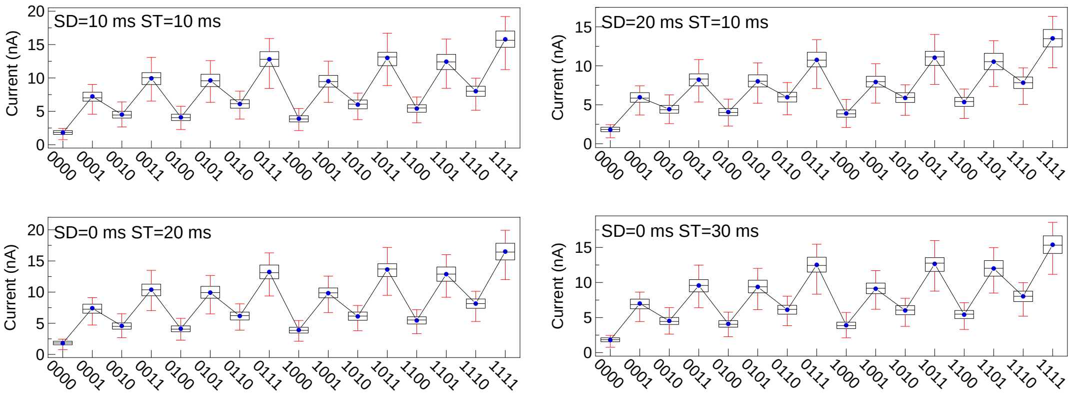

Supplementary Fig. 6 Statistic data of SMP1 of the 16 inputs sampled by different conditions of SD and ST. Each input contains 100 repetitive measurements. The increment of SD and ST causes the decline of SMP1 absolute value. Despite the statistic distribution trend is similar under different conditions, the minimum SD and ST generate the optimal parameters to ditinguish 16 inputs owing to fact that larger absolute current indicates more fault tolerance.

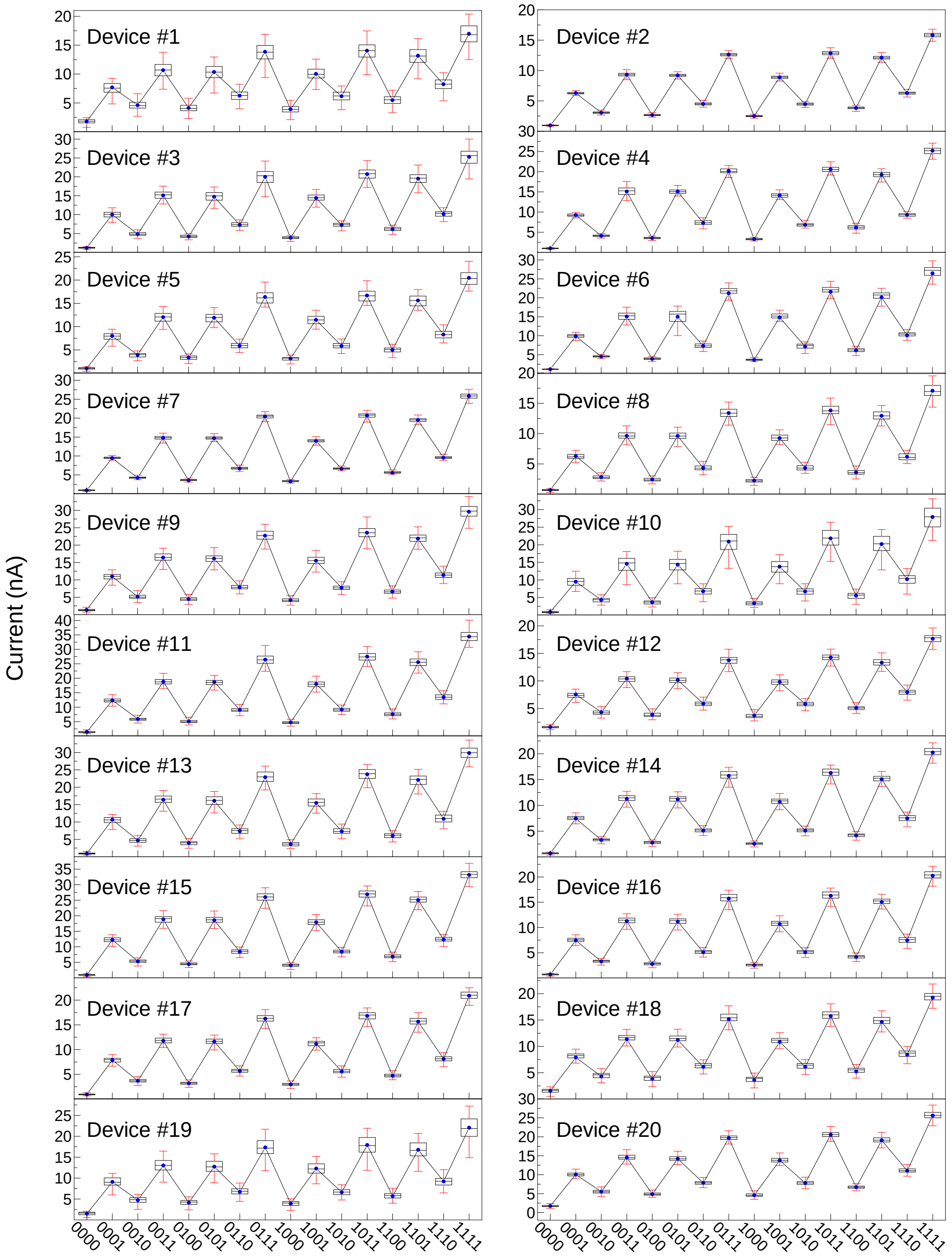

Supplementary Fig. 7 Statistic data of SMP1 of the 16 inputs from stochastically selected 20 a-GaO$_x$ photo-synapse devices. The sampling parameters are fixed at SD=0 ms and ST=10 ms. Each input contains 100 repetitive measurements. A similar distribution trend indicates that all devices exhibit decent classification performances.

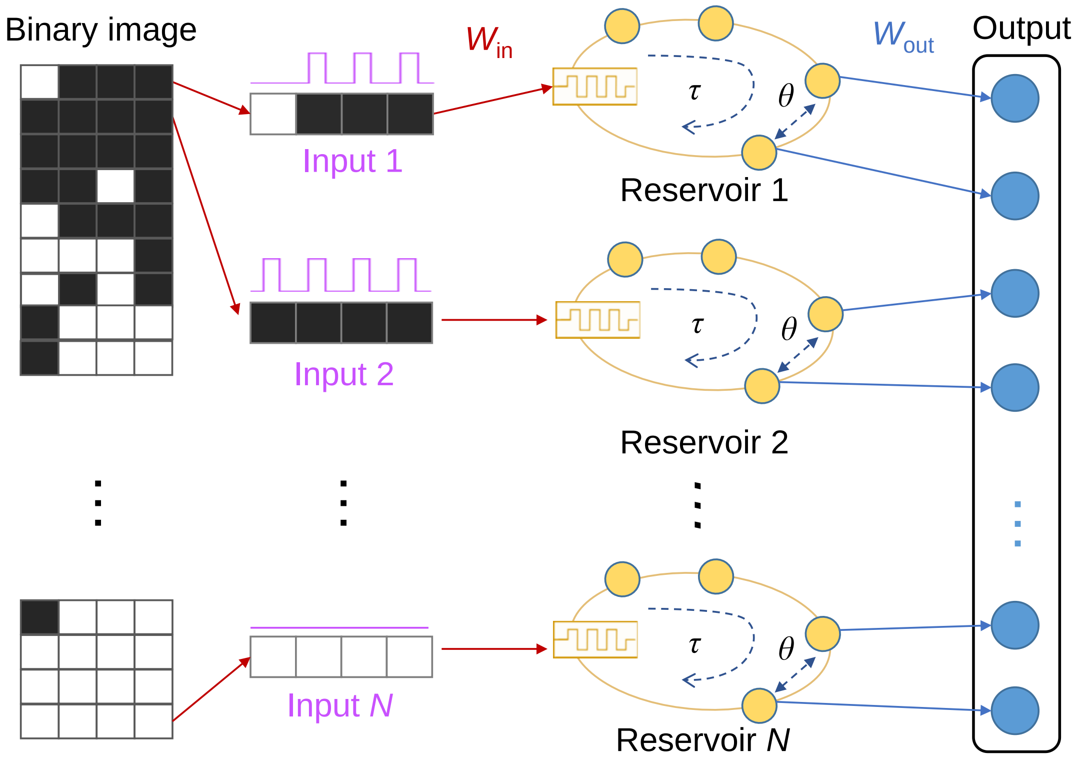

Supplementary Fig. 8 Schematic diagram of the parallel time-delayed reservoir network as a demonstration of our work. The image is divided suitably then input into the reservoirs in parallel. The virtual nodes of each reservoir are coupled with a time interval θ. For the designed readout network, only the last 1 or 2 nodes of each reservoir are utilized to construct the output vector.

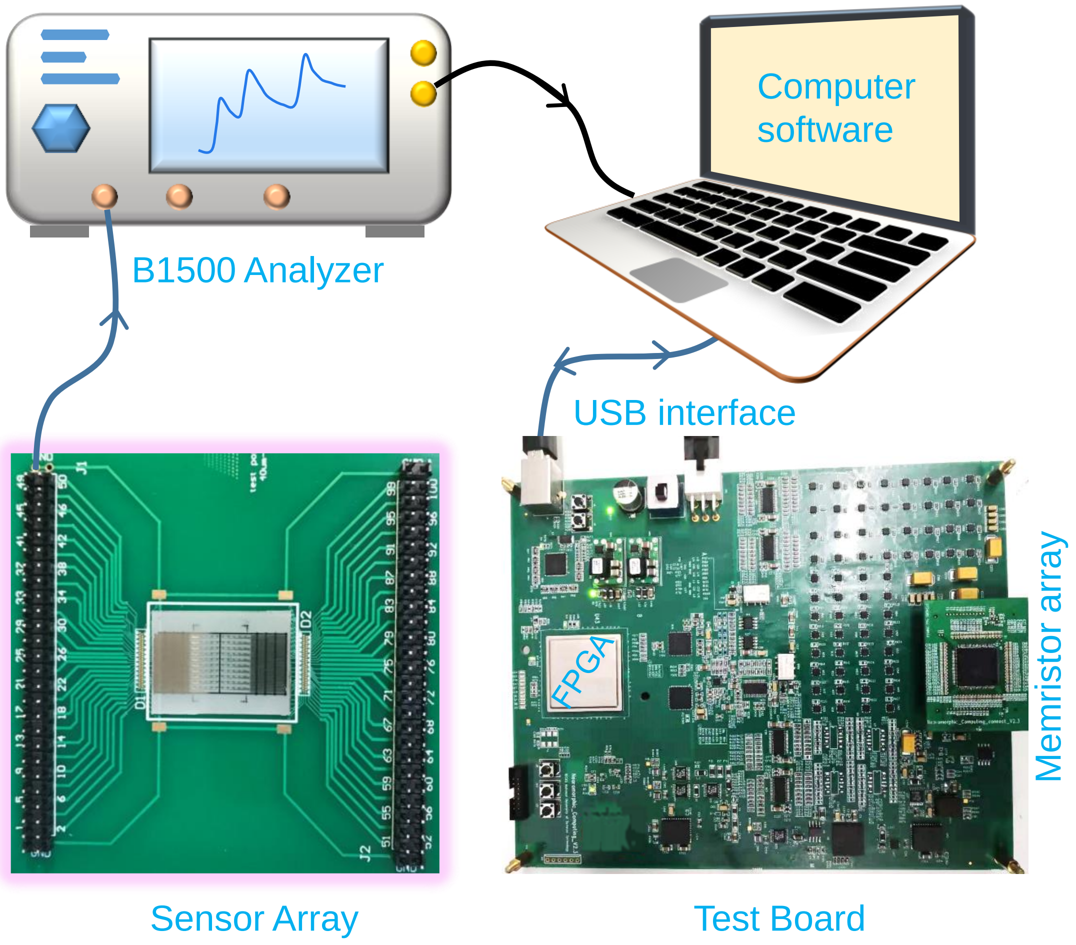

Supplementary Fig. 9 Schematic diagram and photograph of the hardware DUV in-sensor RC system. The system includes a sensor array (photo-synapse reservoir layer), B1500 semiconductor analyzer, a computer with USB data interface, and a readout test board (cosisting of an 1 K-bit memristor array and a FPGA controller).

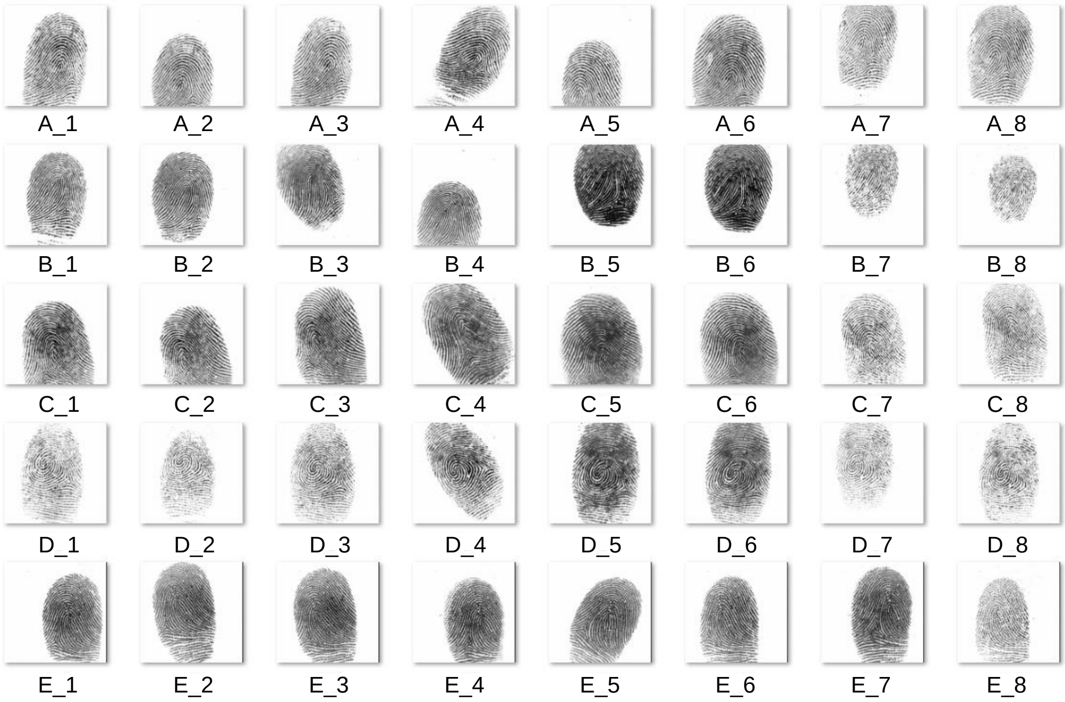

Supplementary Fig. 10 Overview of all the fingerprint images from the FVC 2002 database utilized in this work. Fingerprints are collected from 5 random persons (A-E) and each person takes 8 original fingerprint images.

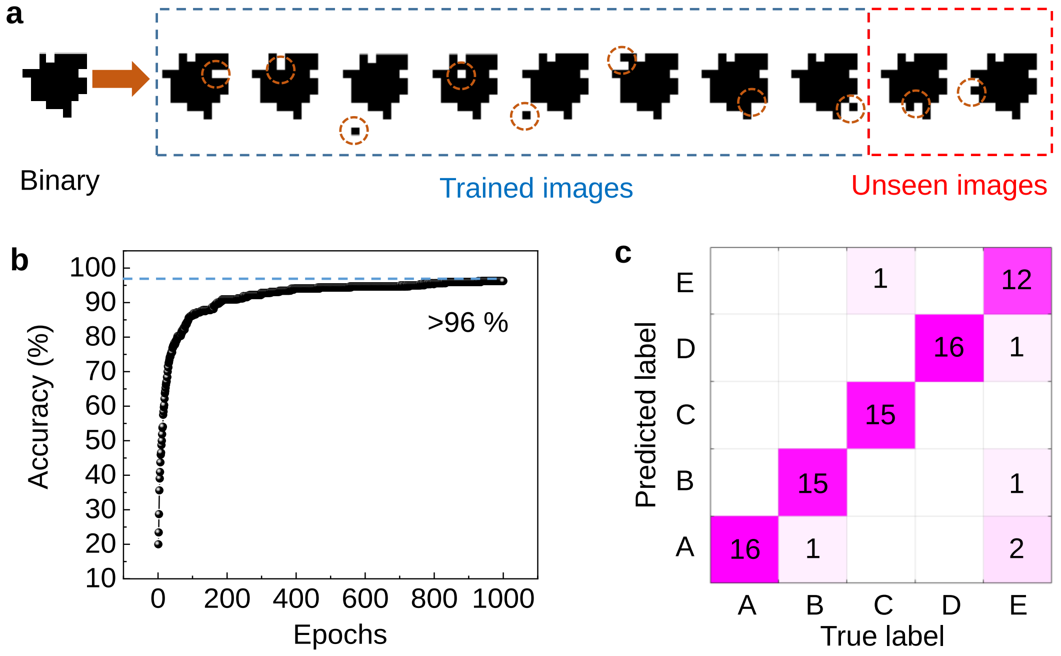

Supplementary Fig. 11 Recognition simulation of the unseen fingerprint images. **a** Expansion of the data set of the fingerprints from 40 to 400 images by introducing one random noise pixel in each original image for 10 times (taking the C-1 image in Supplementary Fig. 10 as an example), owing to the finite scale of the FVC 2002 database. 80% of the fingerprint images were set as the training set and the other 20% as the test set (namely the unseen images). **b** Accuracy convergency of the training process within 1000 epochs. Considerable recognition accuracy can be achieved upon certain training epochs. **c** Confusion matrix of the fingerprint recognition with the unseen images as the test set. The test accuracy is extracted to be 92.5%.

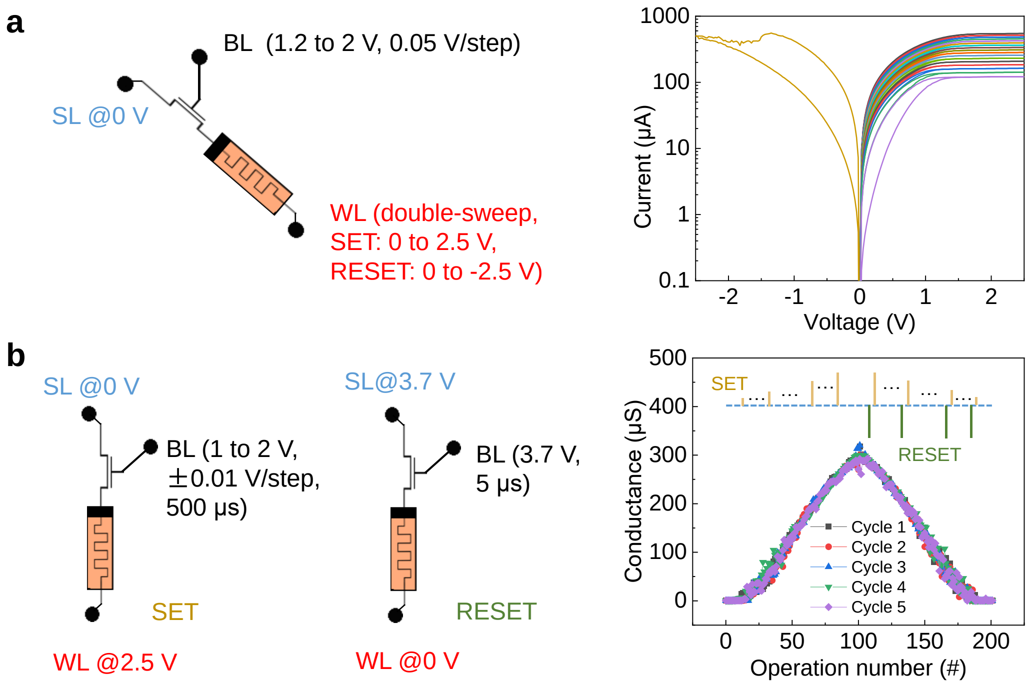

Supplementary Fig. 12 Basic operations and resistance/conductance characteristics of the memristor in the array. a Operation parameters (left) and the I-V characteristics (right) under DC double sweep mode of one typical memristor. When the source line (SL) is grounded and the bit line (BL) is fixed at a certain voltage, the DC voltage on the word line (WL) conducts double-sweep from 0 to 2.5 V to SET and 0 to -2.5 V to RESET. The resistance state can be well modulated by different compliance currents determined by the bias of BL. **b** Operation parameters of the pulse SET (left) and pulse RESET (middle) and the gradual conductance modulation for 5 cycles under successive stimulations (right) of one typical memristor. In the conductivity rising stage, only the pulse SET operations are implemented, in which the bit line voltage increases from 1 to 2 V with a step of 0.01 V. While in the conductivity decline stage, each conductance state is modulated by a couple of pulse RESET and pulse SET: first, a RESET operation is conducted to erase the conductance; then, a pulse SET is applied, in which the bit line voltage decreases from 2 to 1 V with a step of -0.01 V. The conductance value could be repeatatively regulated within approximately 0-300 μS.

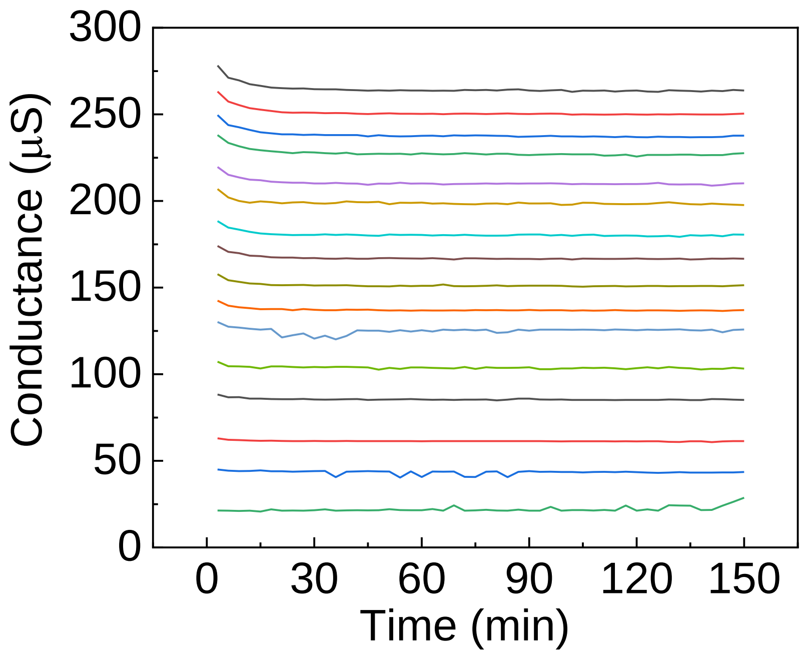

Supplementary Fig. 13 Retention performance of 16 pre-trained conductance states (weights) of one typical memristor in the array. Each conductance state is measured every 3 minutes within 150 minutes. The admirable retention characteristics demonstrate that once the training has been completed, the local memristor array could be used for offline recognition for a long time.
